# The Singlet–Triplet Gap of Pyruvic Acid

**DOI:** 10.1021/jacs.5c13075

**Published:** 2025-10-01

**Authors:** E. Michi Burrow, Javier Carmona-García, Connor J. Clarke, Basile F. E. Curchod, Jan R. R. Verlet

**Affiliations:** † Department of Chemistry, 3057 Durham University, Durham DH1 3LE, United Kingdom; ‡ Centre for Computational Chemistry, School of Chemistry, 1980 University of Bristol, Bristol BS8 1TS, United Kingdom; § J. Heyrovský Institute of Physical Chemistry, Czech Academy of Sciences, Prague 8, 110 00, Czech Republic

## Abstract

Understanding the gas-phase photochemistry of pyruvic acid is crucial to assessing its role and evolution in the atmosphere and relies on knowledge of the relative energy gaps between the atmospherically relevant excited electronic states of the molecule. However, accurate determination of these gaps, particularly between the lowest excited singlet and triplet states, has remained elusive due to the challenge of directly interrogating triplet states of the isolated molecule. In this work, we combine anion photoelectron spectroscopy and computational photochemistry to determine that the adiabatic S_1_-T_1_ energy gap of pyruvic acid is 0.29 ± 0.04 eV. This study provides a reference value for the singlet–triplet energy gap of pyruvic acid and validates an approach that combines theory and experiment to determine energy gaps for volatile organic compounds of atmospheric interest.

## Graphical abstract

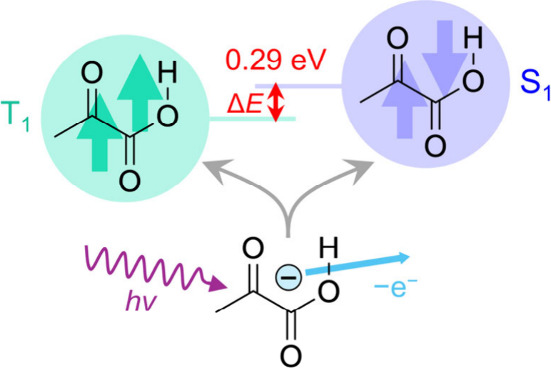

Pyruvic acid (PA) is an atmospherically relevant organic compound, whose reactivity is suggested to contribute to the formation of secondary organic aerosols (SOAs).^1–9^ Given its ubiquitous and multiphasic presence in the atmosphere,^10–14^ the chemistry of PA has been extensively analyzed both experimentally and theoretically.^15–31^ In particular, its photochemistry has received attention as the primary atmospheric sink of PA,^18,32^ with stark differences observed when PA is in the gas phase or in an aqueous environment. In the aqueous phase, PA photochemistry is primarily driven by triplet states,^19,33–37^ while singlet pathways appear to dominate the photochemical reactivity of gaseous PA,^15,16,20,21^ ^,^ ^27,28,31^ although experimental evidence indicates that triplet states may still contribute to the formation of specific photoproducts.^31^ Despite the importance of the interplay between singlet and triplet states in PA, there is a critical lack of information about their relative energy gaps, which are essential to fully mapping the potential photochemical pathways of PA and other keto acids in the gas phase.

Low-lying triplet states of gas-phase PA are difficult to study experimentally. They are optically dark, and, to the best of our knowledge, the only study directly probing triplet excited states of PA in the gas phase used electron energy loss spectroscopy, which has limited resolution.^38^ Additionally, the efficiency of the singlet-driven photochemical pathways of PA^15,16,20,21^ ^,^ ^27,28,31^ limits spectroscopic investigation of its triplet-state reactivity. Atmospheric absorption of UVA light promotes PA into its first excited singlet electronic state S_1_ (Figure 1a), which is of nπ* character in the Franck–Condon region.^20,28^ After passing through a transition state which enables a proton-coupled electron transfer process from the carboxylic moiety to the ketonic oxygen of the molecule, photoexcited PA reaches a minimum-energy conical intersection between S_1_ and the ground state S_0_ (solid gray arrows in Figure 1a).^20,28^ Once in the ground state, the hot system can revert to PA or, predominantly, eject carbon dioxide (CO_2_) and methylhydroxycarbene (MHC).^20,27,28^ Alternative deactivation pathways involving the triplet manifold have been proposed (dashed gray arrows in Figure 1a),^20^ and a recent experimental study by Sauer et al. presented the first direct evidence for these pathways.^31^ Note that the photochemistry of pyruvic acid is completely different from its conjugate base pyruvate, which has recently seen increasing interest.^39,40^

**Figure 1:**
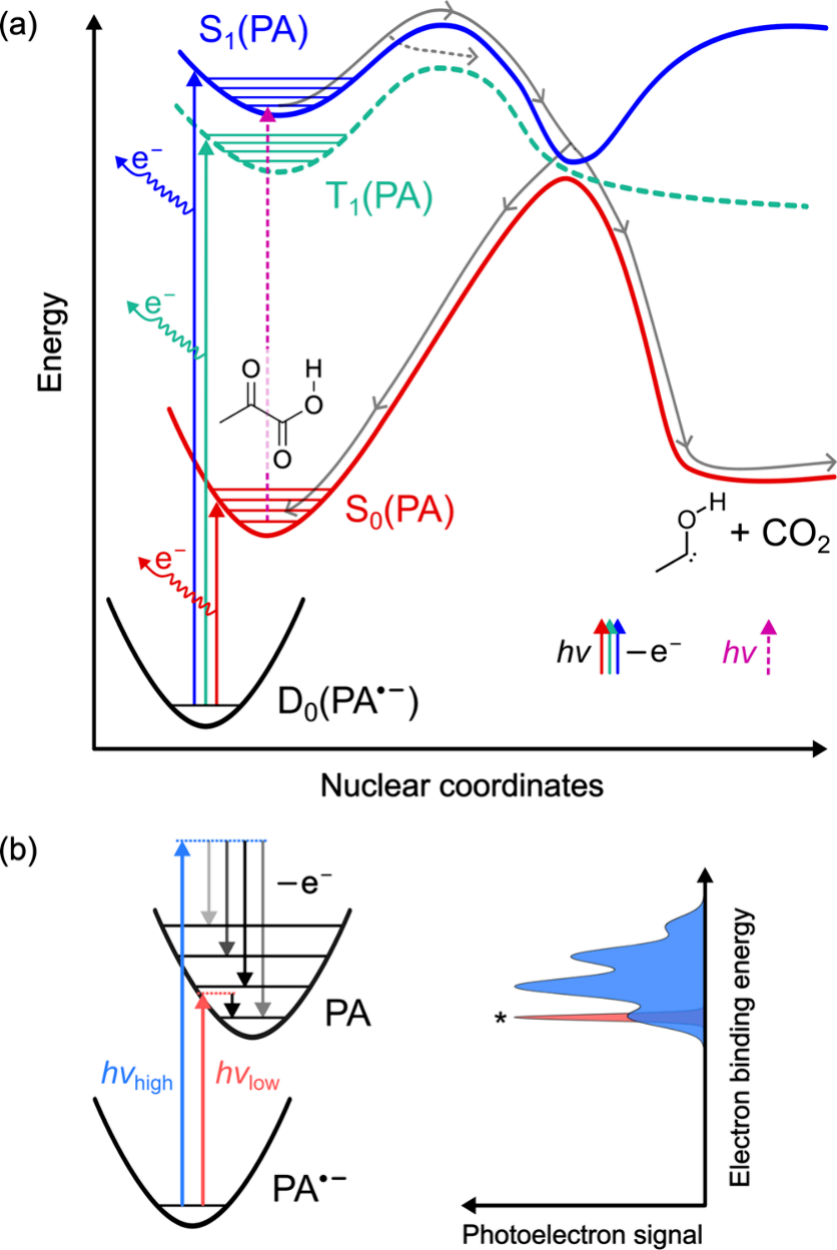
(a) Schematic representation of the deactivation pathways of pyruvic acid (PA) in the gas phase^20,28^ upon absorption of UVA light and the photodetachment of the radical anion of pyruvic acid (PA^•–^) in its doublet ground state (D_0_). Vertical solid arrows show the photodetachment of PA^•–^ and the formation of neutral PA in S_0_, S_1_, or T_1_ upon electron loss. (b) Schematic of anion photoelectron spectroscopy at two different photon energies (*hν*). The asterisk identifies the 0–0 transition, which has higher resolution at lower *hv*.

In this Communication, we present a joint experimental and theoretical study combining anion photoelectron spectroscopy with computational photochemistry to determine the electronic energy gaps and excited-state properties of neutral PA in the gas phase. Photoelectron spectroscopy has relaxed spin selection rules^41^ and can thus be used to measure energy gaps between states of different spin multiplicity.^42,43^ Figure 1a shows how the low-lying triplet and singlet states of PA can be interrogated through photoelectron spectroscopy of its radical anion, PA^•–^, in its doublet ground state D_0_. Irradiation of PA^•–^ with photons of energies *hν* in excess of the electron affinity of the neutral molecule leads to detachment of electrons (Figure 1a). By measuring the kinetic energy of the ejected electrons (eKE), a photoelectron spectrum can be obtained (see the scheme in Figure 1b), which provides information about the electron binding energy (eBE = *hν* – eKE). The measured spectrum varies with *hν* because, with sufficient photon energy, the neutral molecule may be formed in different electronic states and vibrational levels.

The experimental setup has been described in detail previously.^44^ Briefly, gas-phase PA^•–^ was produced via electron attachment to a molecular beam. PA^•–^ was mass-selected before being excited at a range of *hv*, and the subsequently detached electrons were monitored using an imaging photoelectron spectrometer (see Supporting Information for more experimental details).

The photoelectron spectrum encodes information regarding differences in the geometry and electronic structure of the anion and states of the neutral system. For instance, by measuring adiabatic detachment energies (ADE) between the D_0_ anion ground state and different electronic states of the neutral molecule, excitation energies between the neutral states can be obtained. Complementary computed photoelectron spectra are crucial for properly ascribing the different peaks to the corresponding states of the neutral system and mapping the measured vibrational structure to the dominant normal modes excited upon electron loss. In this work, the calculated vibrationally resolved spectra were obtained by employing the vertical Hessian model within the harmonic approximation, using the time-independent approach,^45–47^ based on electronic-structure quantities obtained with density functional theory (DFT) and linear-response time-dependent DFT within the Tamm–Dancoff approximation.^48^ See Supporting Information for the computational details and a benchmark of the methodology.

Two photoelectron spectra of PA^•–^ measured at *hν* = 2.50 and 4.66 eV are shown in Figure 2. We first focus on the spectrum acquired at *hν* = 2.50 eV (Figure 2a, left), which exhibits a broad, structured direct photodetachment feature, corresponding to the D_0_ → S_0_ transition. The ADE can be extracted from the eBE of the 0–0 transition (positioned at the lowest eBE), although this peak has a limited spectral resolution. As suggested in Figure 1b, a more accurate determination of the ADE can be made by setting *hν* to be just above the threshold for photodetachment.^49,50^ From an additional photoelectron spectrum acquired at *hν* = 0.925 eV (Figure 2c, see Figure S1 for more details), we establish that ADE­(D_0_ → S_0_) = 0.901 ± 0.005 eV. This direct measurement shows the electron affinity of PA to be slightly higher than the 0.84 ± 0.02 eV value previously reported.^51^

**Figure 2:**
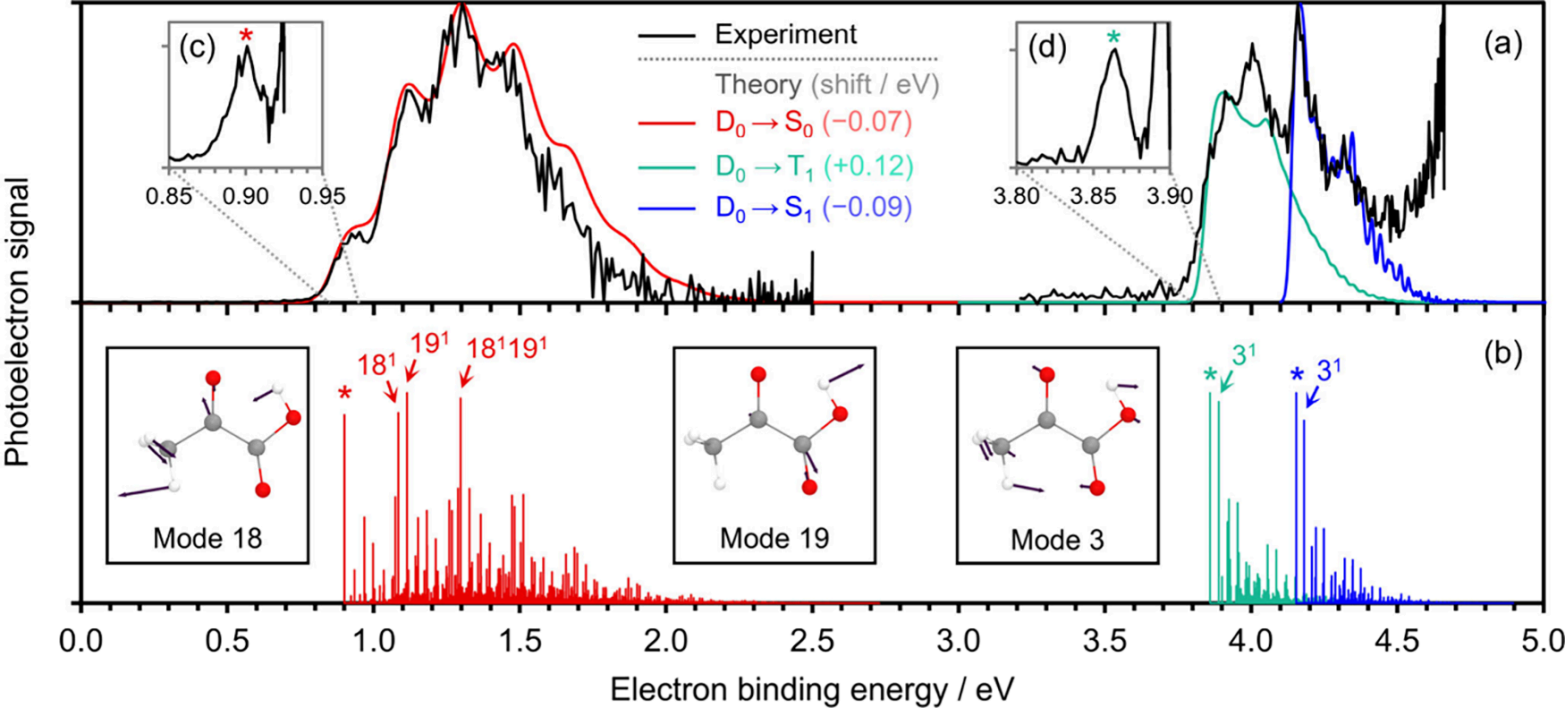
(a) Experimental photoelectron spectrum of PA^•–^ for *hν* = 2.50 (left) and 4.66 eV (right) in black. (b) Calculated vibrationally resolved photoelectron spectra for the D_0_ → S_0_, D_0_ → T_1_, and D_0_ → S_1_ transitions of PA^•–^ are shown in red, green, and blue, respectively, as stick spectra. Calculated spectra were shifted to align with the experimental spectra. Included in (a) are the calculated spectra convolved with experimental resolution. Highly contributing transitions are identified with a label n^y^ where n is the vibrational mode of the corresponding state of the neutral system, and y is the excited quanta.^47^ Relevant normal modes of PA are shown with arrows representing atomic displacements. The inset panels show photoelectron spectra acquired using *hν* slightly above the 0–0 transition energy for (c) D_0_ → S_0_ and (d) D_0_ → T_1_. The asterisk highlights the peak assigned to the 0–0 adiabatic detachment energy.

The assignment of the experimental D_0_ → S_0_ transition is supported by the corresponding calculated photoelectron spectrum (Figure 2b, left) from which an eBE of 0.97 eV for the 0–0 transition is extracted. To better compare the calculated and experimental photoelectron signals, the calculated spectrum has been shifted by −0.07 eV to match the eBE of the experimental 0–0 transition, and each calculated stick transition was convolved with a Gaussian function of width matching the experimental resolution (Supporting Information). The calculated and experimental spectra are in excellent agreement for *hν* = 2.50 eV (see Figure 2a), particularly on the low-eBE side.

Analysis of the calculated vibrational progressions indicates that the measured vibrational structure around the 0–0 region and the maximum is mainly dominated by excitations of normal modes 18 and 19 of final state S_0_ (Figure 2b, left). The associated molecular displacements predominantly involve the stretching of the carbon–oxygen double bonds of the ketonic and carboxylic moieties for modes 18 and 19, respectively. The C=O stretching is accompanied by displacements of the hydrogen atom from the hydroxyl group in both modes, as well as of the carbon–hydrogen bonds in mode 18.

The photoelectron spectrum acquired at *hν* = 4.66 eV (Figure 2a, right) also shows vibrationally resolved features. Photodetachment to both of the neutral excited states, S_1_ and T_1_, is likely to be accessible at this *hν* (see Table S1), but it is difficult to discern which features correspond to which transitions from the measured spectrum alone. The T_1_ state is expected to be lower in energy, and therefore the low-eBE onset of the photoelectron signal should correspond to the 0–0 transition of D_0_ → T_1_. This ascription is supported by the calculated spectrum, which predicts an eBE of 3.74 eV for the 0–0 transition. We again acquired an additional near-threshold (*hν* = 3.90 eV) photoelectron spectrum (Figure 2(d), see Figure S1 for more details), allowing the determination of ADE­(D_0_ → T_1_) = 3.863 ± 0.005 eV. Note that the calculated spectrum in Figure 2 has been shifted by +0.12 eV, and the intensity of the convolved spectrum has been rescaled to match that of the experimental photoelectron signal for the 0–0 transition. The agreement between the theoretical and experimental spectra for the D_0_ → S_0_ and D_0_ → T_1_ transitions enables a reliable assignment of the higher eBE part of the *hν* = 4.66 eV spectrum to the D_0_ → S_1_ transition, based on the corresponding calculated spectrum (Figure 2a,b, right). We extract from the results presented in Figure 2a that the experimental ADE­(D_0_ → S_1_) = 4.15 ± 0.03 eV, fairly consistent with the calculated eBE for the 0–0 transition being 4.24 eV. Near-threshold spectra for D_0_ → S_1_ displayed no clear 0–0 transition due to a competing signal associated with D_0_ → T_1_. The peak at eBE = 4.00 eV that is unaccounted for by the calculated spectrum is likely due to an excited state of the anion leading to autodetachment.^52,53^

In the measured vibrational fine structure of the spectrum at *hν* = 4.66 eV, the highest intensity peak around the 0–0 region for the D_0_ → S_1_ and D_0_ → T_1_ transitions corresponds to excitations of a bending mode of the corresponding neutral states which brings the −OH group toward the ketone (normal mode 3 in Figure 2b, right). This normal mode is likely involved in the proton transfer process that leads to decarboxylation. The closeness between the vibronic progressions of the two transitions reflects that the potential energy surfaces of the S_1_ and T_1_ states around the Franck–Condon region are similar. This observation is further supported by the electronic states sharing nπ* character with only slight geometrical differences between their minimum structures (Figures S3 and S4).

By appropriately subtracting the ADEs reported here, we can extract the term energies of the S_1_ and T_1_ states of neutral PA: the S_1_ term energy *E*_S1_ = 3.25 ± 0.03 eV, close to a previously reported value of 3.312 eV^54^ obtained by photofragment yield spectroscopy; the T_1_ term energy *E*_T1_ = 2.962 ± 0.008 eV, which has elsewise proven difficult to determine spectroscopically.^38^ Our measured value is fairly consistent with earlier computational predictions.^20,27^ By determining both *E*_S1_ and *E*_T1_ of PA, we deduce the S_1_-T_1_ energy gap to be 0.29 ± 0.04 eV. Past calculated values have typically been close to, but slightly below, our measured value (e.g., 0.23 eV,^20^ 0.26 eV^27^). Although this small energy gap suggests that intersystem crossing from S_1_ to T_1_ can efficiently occur around the Franck–Condon region of PA, this process appears to be secondary considering the calculated low spin–orbit coupling between both electronic states^28^ given their similar character and in line with the El-Sayed rule. Note that we can expect a similar S_1_-T_1_ energy gap in the aqueous phase as solvent effects should influence the stability of both electronic states on the same footing, given their common nπ* character in the Franck–Condon region of PA.

In conclusion, we showed that combining photoelectron spectroscopy with computational photochemistry allows the determination of energy gaps, including optically inaccessible (excited) electronic states for volatile organic compounds of atmospheric interest. The synergy between theory and experiment allowed us to accurately determine the energy gap between the S_1_ and T_1_ excited states of gas-phase PA, 0.29 ± 0.04 eV, a quantity that has remained elusive until now. This S_1_-T_1_ energy gap may serve as a reference value for further work that hopes to elucidate the extent to which the triplet state is involved in the photochemistry of PA, as well as why the light-induced reactivity of PA changes significantly from the gas phase to an aqueous environment. The multipronged strategy introduced here can be applied to other atmospherically relevant organic compounds and will be generalized in the future to study the effect of microsolvation on the photochemistry of these molecules.

## Supplementary Material



## Notes

Extended experimental and computational details, near-threshold photoelectron spectra, validation of the computational methods, molecular structures of the different minima, character of excited states based on natural transition orbitals and frontier Kohn–Sham orbitals, including Figures S1–S7 and Table S1 (PDF)

## Abbreviations

PA: Pyruvic acid
SOA: Secondary organic aerosol
MHC: Methylhydroxycarbene
ADE: Adiabatic detachment energy
DFT: Density functional theory
