# Supplementary material for: The Singlet–Triplet Gap of Pyruvic Acid
Source: J Am Chem Soc. 2025 Oct 1;147(41):36987–91. doi: 10.1021/jacs.5c13075 (PMC12532293; doi:10.1021/jacs.5c13075)
Supplement: Supplementary file 1 [file ja5c13075_si_001.pdf]

Supporting Information:

The Singlet-Triplet Gap of Pyruvic Acid

E. Michi Burrow[a,‡], Javier Carmona-García[b,‡], Connor J. Clarke[a,‡], Basile F. E. Curchod[b,*], and Jan R. R. Verlet[a,c*]

[a]*Department of Chemistry, Durham University, Durham DH1 3LE, United Kingdom*

[b]*Centre for Computational Chemistry, School of Chemistry, University of Bristol, Bristol BS8 1TS, United Kingdom*

[c]*J. Heyrovský Institute of Physical Chemistry, Czech Academy of Sciences, Prague 8, Czech Republic*

‡These authors contributed equally.

*Corresponding authors.

Email: basile.curchod@bristol.ac.uk, j.r.r.verlet@durham.ac.uk

Table of contents

1. Experimental set up 2

2. Near-threshold photoelectron spectra 2

3. Broadening of stick spectra 4

4. Extended computational details 5

5. Electronic structure methods' validation 6

6. Validation of the Vertical Hessian (VH) model 7

7. Validation of the Time-Independent (TI) formulation 11

8. References 12

1. Experimental set up

The experimental set up has been described in detail previously.[1] Briefly, pyruvic acid (PA) vapor was seeded into an argon backing gas line held at a pressure of 10 bar, which was expanded into ultrahigh vacuum via a pulsed Even-Lavie valve.[2] The supersonic expansion passed through a ring filament ionizer, generating pyruvic acid radical anions, ($PA^{\bullet-}$). These were isolated and mass-selected via time-of-flight mass spectrometry.[3] $PA^{\bullet-}$ was intersected with the tunable wavelength output of a nanosecond Nd:YAG-pumped optical parametric oscillator. The resultant photoelectrons were collected and measured using a velocity map imaging detector. Spectra were obtained using the polar onion peeling (POP) algorithm.[4] The resolution of the spectra was ~3% of the outgoing eKE and was calibrated to the well-known spin-orbit splitting of atomic iodide.[5] The raw experimental data can be found on Zenodo, doi: 10.5281/zenodo.17225039.

2. Near-threshold photoelectron spectra

To better extract the energies of the 0-0 transitions associated with $D_0 \longrightarrow S_0$ and $D_0 \longrightarrow T_1$, we acquired additional photoelectron spectra at photon energies ($h\nu$) slightly above the threshold for photodetachment. Resultantly, electrons arising from the origin transitions were released with very low eKE, allowing the velocity map imaging spectrometer to be operated at high resolution (~ 1 meV and below). The spectra are shown in Figure S1, each demonstrating a single clear peak

at the expected electron binding energy (eBE) of the 0-0 transition. Simple Gaussian fits were applied to aid extraction of the 0-0 transition energies.

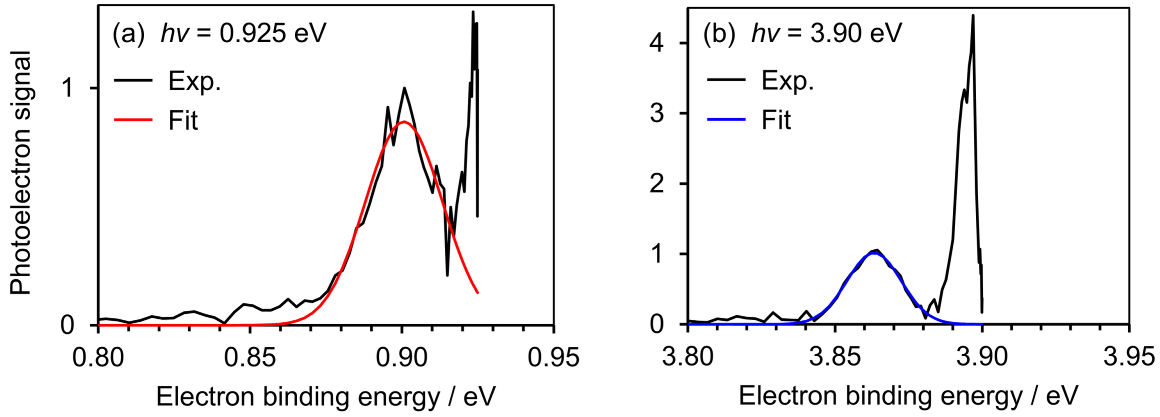

Figure S1. Photoelectron spectra acquired using photon energies *hv* slightly above the 0-0 transition energy for (a) $D_0 \longrightarrow S_0$ and (b) $D_0 \longrightarrow T_1$. Spectra are normalized to the peak of the origin transitions. The red and blue lines represent Gaussian fits to the 0-0 transitions.

The stated uncertainties (5 meV) in the extracted eBEs primarily arose from the calibration of the OPO laser output, rather than the photoelectron spectrometer resolution. We also checked whether the choice of image reconstruction algorithm (POP) was significant; a second algorithm, MELEXIR,[6] was applied and produced consistent transition energies.

No near-threshold photoelectron spectrum was acquired for the $D_0 \longrightarrow S_1$ transition, due to competing photoelectron signal arising from the $D_0 \longrightarrow T_1$ transition at photon energies near hv = 4.16 eV. In particular, the near-threshold $D_0 \longrightarrow S_1$ signal has comparably little intensity in accord with the Wigner threshold law,[7] and thus we found that using a higher photon energy provided a far better determination. The stated uncertainty in the $D_0 \longrightarrow S_1$ energy (30 meV) was dominated by the limited resolution of the electron spectrometer, since electrons arising from the 0-0 transition were ejected with a higher eKE.

3. Broadening of stick spectra

Photoelectron spectra of $PA^{\bullet-}$ (at $h\nu$ = 2.50 and 4.66 eV) contained vibrational structure, with a resolution limited by the velocity map imaging spectrometer. To better compare the computed stick spectra to the experimental results, we applied an eBE-shift to the sticks such that the 0-0 transition aligned with the measured value, then convolved each stick with a Gaussian function of the same width as the experimental spectral resolution. Since the resolution of the spectrometer is dependent on the eKE (being ~3% of the outgoing energy), the Gaussian linewidth was different for each transition, with sticks positioned at lower eBE (i.e. higher eKE) being broadened to a greater extent. For each electronic transition, the resulting broadened sticks were summed to produce a spectrum comparable to experiment.

In Figure 2a, the intensity of the convolved calculated photoelectron spectrum of $D_0 \longrightarrow T_1$ was scaled (to 70%) such that its maximum matched the height of the corresponding experimental 0-0 transition. This arbitrary intensity scaling was chosen for presentation purposes; we wanted to clearly distinguish between the calculated photoelectron signal associated with the different excited states, and illustrate how the calculated vibrational progression of the $D_0 \longrightarrow T_1$ transition compares with the experimentally measured structure. Therefore, the scaling does *not* reflect physical properties associated with the transition (e.g. relative photodetachment cross-sections).

Alternatively, the convolved calculated photoelectron spectra associated with the T_1 and S_1 excited states could be combined into a single spectrum, to be compared with the experiment. We demonstrate this result in Figure S2, overlaid with the experimental spectrum. For simplicity, the stick spectra were individually normalized (to the highest intensity stick, i.e. resembling the spectra in Figure 2b) prior to their simultaneous convolution. The resulting convolved spectrum was then normalized to its highest intensity, as was the experimental spectrum. The agreement between the two is good, comparable to that shown in Figure 2a.

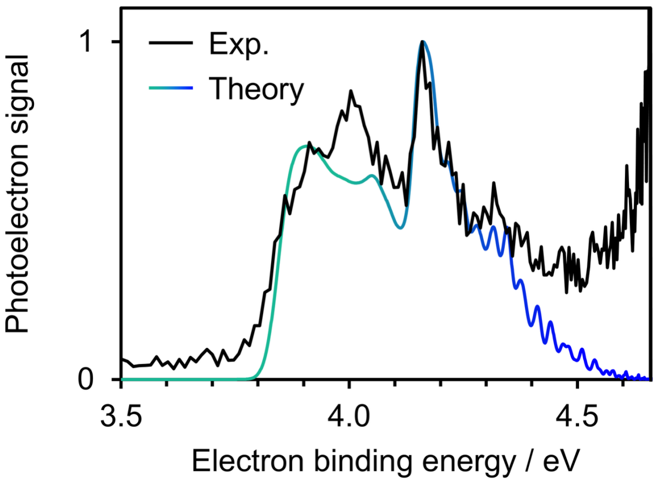

Figure S2. Photoelectron spectrum acquired using *hv* = 4.66 eV (black), together with a computed spectrum, produced via the simultaneous convolution of a Gaussian function with the $D_0 \longrightarrow T_1$ and $D_0 \longrightarrow S_1$ stick spectra shown in Figure 2b.

4. Extended computational details

The vibrationally resolved spectra were calculated with the vertical hessian (VH) model within the harmonic approximation, using the sum-over-states or the so-called time-independent (TI) approach, which allows for the characterization of the individual vibronic transitions that conform the spectra, extracting the contributions of each normal mode to each individual transition[8-10], as implemented in FCclasses3.[9] All calculations were carried out at 0 K, considering only Franck-Condon (FC) terms, and employing curvilinear internal coordinates (namely valence internal coordinates) for the computation of normal modes both in the initial and final states. This approach requires the obtention of the Hessian of the initial and final states, that is, the ground state D_0 of $PA^{\bullet-}$ and the neutral states of PA, computed at the optimized ground-state geometry of $PA^{\bullet-}$. The optimization and Hessian calculations were carried out using density functional theory (DFT) with the ωB97X-D functional[11] and the aug-cc-pVDZ[12] basis set for D_0, S_0, and T_1. For the S_1 state, linear-response time-dependent density functional theory (LR-TDDFT) was employed within the Tamm–Dancoff approximation (TDA), and 20 singlet states were computed using the same functional and basis set. All DFT and LR-TDDFT/TDA calculations were performed with Gaussian16 C.01.[13] The computed stick spectra and the ground-state optimized structure of $PA^{\bullet-}$ can be found on Zenodo, doi: 10.5281/zenodo.17225039.

5. Validation of the electronic structure methods

The use of the (U)DFT/ωB97XD/aug-cc-pVDZ and LR-TDDFT/TDA/ωB97XD/aug-cc-pVDZ levels of theory was validated by comparing the calculated vertical ionization energies, that is, the electron binding energy for each transition calculated at the ground-state optimized structure of $PA^{\bullet-}$, with the energies obtained with the explicitly correlated coupled cluster singles and doubles method approximation as proposed by Hättig, Tew, and Köhnwith, with perturbative triples corrections (CCSD(T)(F12*)),[14, 15] and equation of motion coupled-cluster singles and doubles for ionization potentials variant (IP-EOM-CCSD). In both cases, the aug-cc-pVTZ basis set was employed, and 10 states were computed for the IP-EOM-CCSD calculations. As shown in Table S1, the DFT and LR-TDDFT/TDA are in good agreement with the coupled-cluster predictions considering the smaller basis set employed in the former calculations and the fact that the initial state is a negatively charged doublet state. The vertical ionization energies for the $D_0 \longrightarrow S_0$ and $D_0 \longrightarrow S_1$ transitions are overestimated with DFT and LR-TDDFT/TDA, respectively, which justifies the small negative energy shifts applied to the calculated spectra (see Figure 2 of the main text) so that the eBE of the calculated 0-0 transitions coincide with the experimentally determined value. In the case of the $D_0 \longrightarrow T_1$, the predicted vertical ionization energy obtained with DFT is underestimated, validating the applied small positive energy shift to the calculated spectrum. The CCSD(T)(F12*) and IP-EOM-CCSD calculations were performed with Turbomole 7.4.1[16] and ORCA 6.0.0,[17, 18] respectively.

Table S1. Vertical ionisation energies for the $D_0 \longrightarrow S_0$, $D_0 \longrightarrow T_1$, and $D_0 \longrightarrow S_1$ transitions calculated with (U)DFT, LR-TDDFT/TDA, (U)CCSD(T)(F12*), and IP-EOM-CCSD for the optimized ground-state geometry of $PA^{\bullet-}$ obtained with UDFT/ωB97X-D/aug-cc-pVDZ.

**Method**	**$D_0 \longrightarrow S_0$**	**$D_0 \longrightarrow T_1$**	**$D_0 \longrightarrow S_1$**
(U)DFT/ωB97XD/aug-cc-pVDZ	1.498	4.018	-
(U)DFT and LR-TDDFT/TDA/ωB97XD/aug-cc-pVDZ	-	-	4.475
(U)CCSD(T)(F12*)/aug-cc-pVTZ	1.318	4.252	-
IP-EOM-CCSD/aug-cc-pVTZ	1.314	4.046	4.241

6. Validation of the Vertical Hessian (VH) model

The VH model employed here is better suited than the adiabatic hessian (AH) approach for molecular systems that exhibit a significant structural change between the equilibrium geometry of their initial and final states. In this case, as shown in Figure S3, the S_0 minimum geometry of PA has its $–CH_3$ group rotated with respect to the D_0 minimum geometry of $PA^{\bullet-}$, impeding the application of the AH model. However, in the VH model the Hessian of the final state is calculated at a non-stationary point of the potential energy surface (PES) and imaginary frequencies can arise[8-10]. For the S_0 state, an imaginary frequency associated with the rotation of the $-CH_3$ group rotation is obtained within the normal mode calculation of FCclasses3. After analyzing that this normal mode does not have a significant impact on the spectrum lineshape by turning the imaginary frequency into real and testing different values[9] (see Figure S5a), we removed the internal coordinate associated with this rotation (i.e., the linear combination of all the dihedral angles that define the rotation) for the final calculations. Figure S5b shows validation of this reduced-dimensionality model.

In the case of the T_1 and S_1 states of PA, their minimum geometries, also shown in Figure S3, are very similar because both electronic states have the same $n\pi^*$ character (Figure S4). In addition, as both geometries resemble the D_0 minimum geometry of $PA^{\bullet-}$, including the position of the $–CH_3$ group, the AH model may be applied for further validation of the VH model (see Figure S6) considering that the small differences observed, which may be associated with the anharmonicity of the final state PES,[9, 10] do not affect the qualitative analysis carried out in this work to ascribe the experimental photoelectron signals to the different transitions.

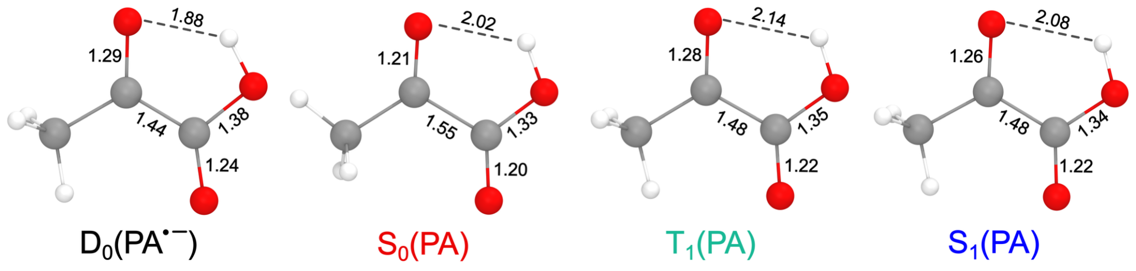

Figure S3. Molecular structures of the D_0 minimum of $PA^{\bullet-}$, and the S_0, T_1, and S_1 minima of PA optimized with (U)DFT (D_0, S_0, T_1) and LR-TDDFT/TDA (S_1) using the ωB97X-D functional and the aug-cc-pVDZ basis set. Note the rotation of the $-CH_3$ group of the S_0 minimum with respect to the D_0, T_1, and S_1 equilibrium structures.

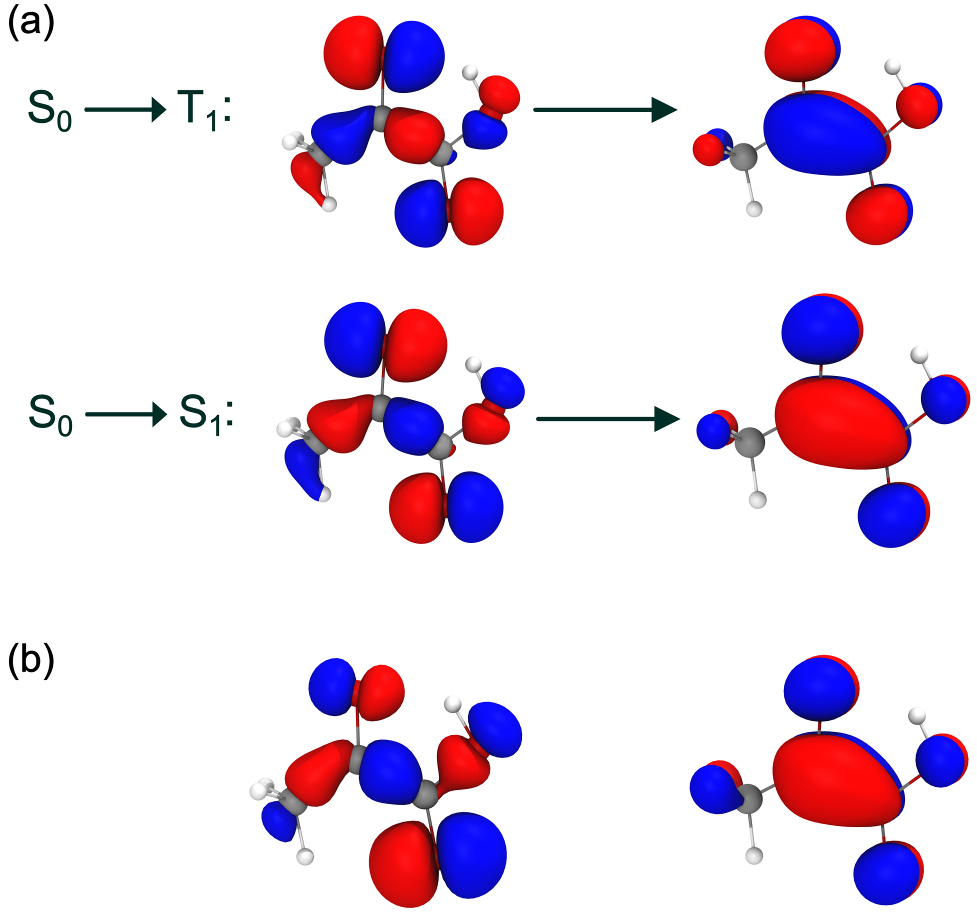

Figure S4. (a) Natural transition orbitals describing the $S_0 \longrightarrow T_1$ and $S_0 \longrightarrow S_1$ excitations of neutral PA obtained with LR-TDDFT/TDA/ωB97X-D/aug-cc-pVDZ. (b) Frontier Kohn–Sham molecular orbitals α-HOMO-1 (left) and α-HOMO (right) of the T_1 state of PA obtained with UDFT/ωB97X-D/aug-cc-pVDZ. All electronic structure calculations in (a) and (b) were carried out on the ground-state optimized geometry of $PA^{\bullet-}$ obtained at the (U)DFT/ωB97X-D/aug-cc-pVDZ level of theory.

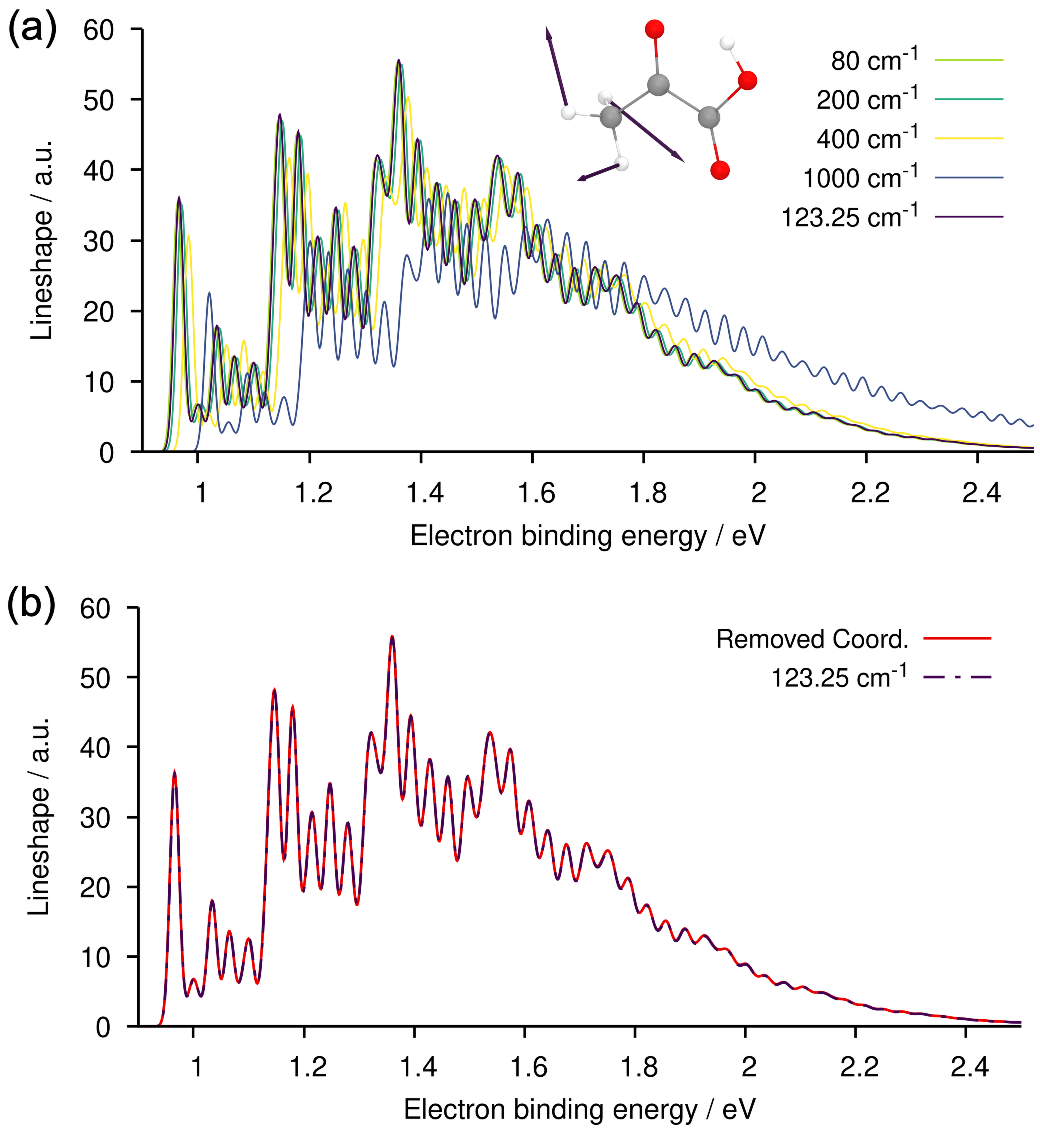

Figure S5. (a) Calculated photoelectron spectra for the $D_0 \longrightarrow S_0$ transition of $PA^{\bullet-}$ setting the imaginary frequency related to the rotation of the $–CH_3$ group to different real values. The corresponding normal mode is shown as an inset, with arrows representing the atomic displacements. The value of 123.25 cm^{-1} corresponds to the original imaginary value turned to a real value. As can be seen, the effect of this normal mode on the spectrum is not significant unless we consider unrealistically high values. (b) Comparison between the spectra obtained by removing the internal coordinate associated with the rotation and with the original imaginary frequency turned into real. Both strategies give similar results, confirming the negligible impact of this internal coordinate and associated normal mode on the final lineshape. The spectra are obtained at 0 K with the (U)DFT/ωB97X-D/aug-cc-pVDZ level of theory, using the VH model in its TI formalism, and convolving each individual transition with a Gaussian function having a half-width at half maximum (HWHM) of 0.01 eV.

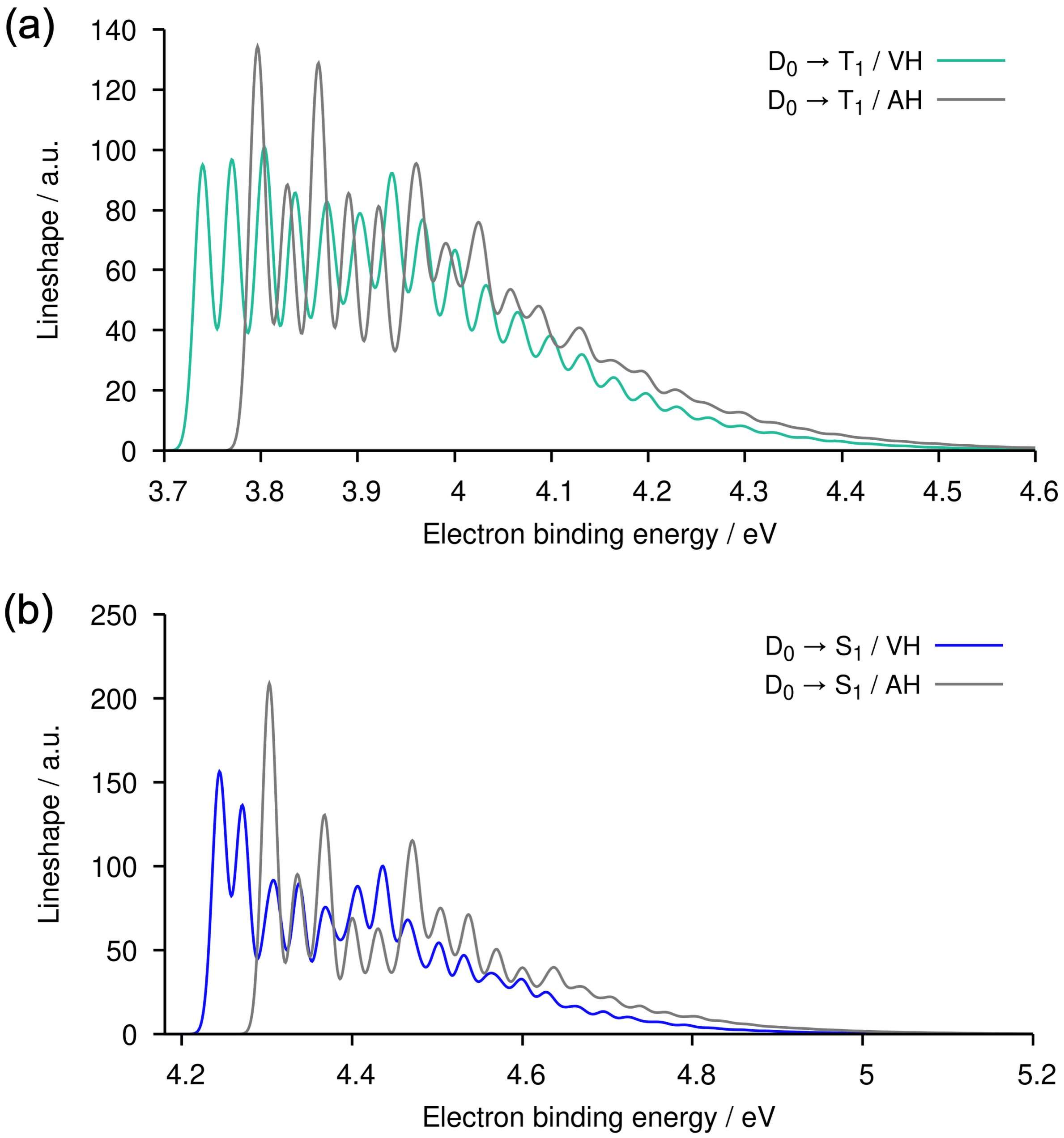

Figure S6. Calculated photoelectron spectra for the $D_0 \longrightarrow T_1$ (a) and $D_0 \longrightarrow S_1$ transitions of $PA^{\bullet-}$ using the vertical hessian (VH) and adiabatic hessian (AH) approximations. Both approaches give similar spectral shapes but slightly shifted (< 0.1 eV) and with small differences in intensity in some of the peaks. Note that these differences do not change the ascription of the experimental photoelectron signals done in the main manuscript. The spectra are obtained at 0K with the (U)DFT/ωB97X-D/aug-cc-pVDZ level of theory for T_1, and LR-TDDFT/TDA/ωB97X-D/aug-cc-pVDZ for S_1, convolving each individual transition with a Gaussian function having a half-width at half maximum (HWHM) of 0.01 eV.

7. Validation of the Time-Independent (TI) formulation

The validation of the usage of the TI framework can be seen in Figure S7 by comparing with the spectra obtained with the Time-Dependent (TD) formulation, which gives the fully converged spectrum at any temperature but does not provide information about the individual transitions.[9] In all cases, the recovery of the total intensity is more than 98% with the TI calculations.

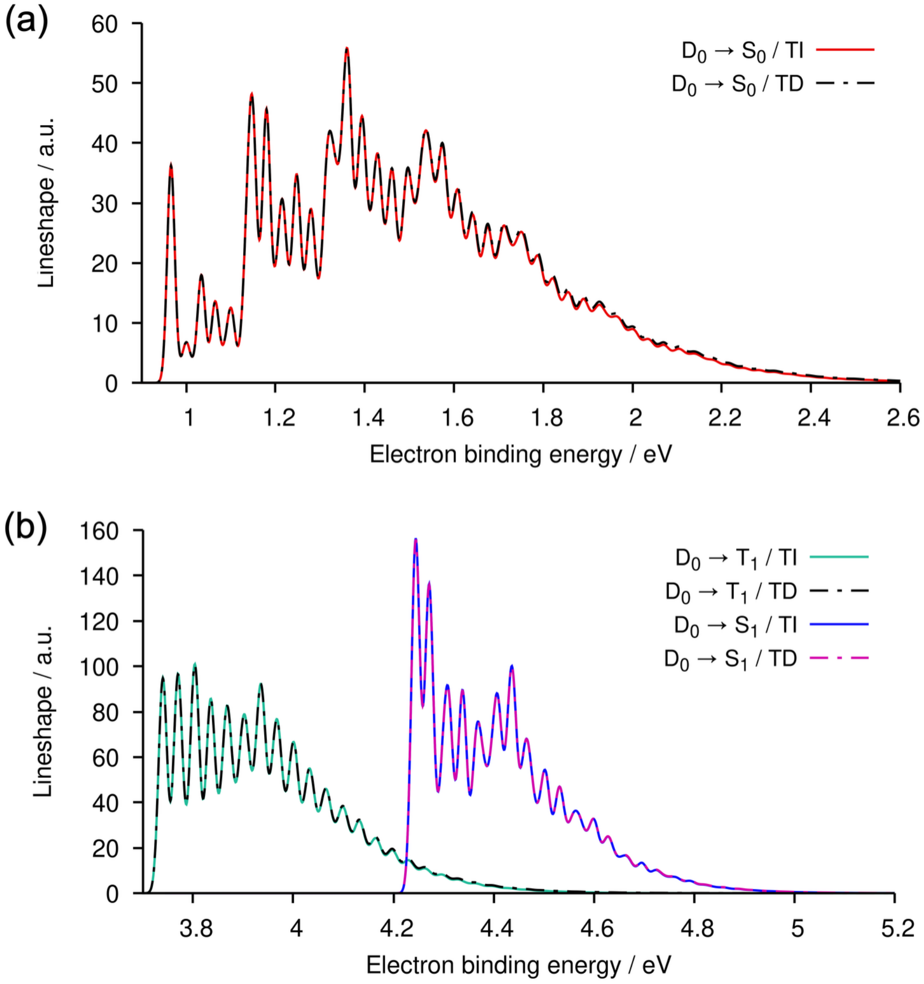

Figure S7. Calculated photoelectron spectra for the $D_0 \longrightarrow S_0$ (a), and $D_0 \longrightarrow T_1$ and $D_0 \longrightarrow S_1$ (b) transitions of $PA^{\bullet-}$ using the time-independent (TI) and time-dependent (TD) formulations. As can be seen, the spectra for all transitions with both models are virtually the same. The spectra are obtained using the vertical hessian (VH) approximation at 0K with the (U)DFT/ωB97X-D/aug-cc-pVDZ level of theory for S_0 and T_1, and LR-TDDFT/TDA/ωB97X-D/aug-cc-pVDZ for S_1, convolving each individual transition with a Gaussian function having a half-width at half maximum (HWHM) of 0.01 eV.

8. References

(1) Rogers, J. P.; Anstöter, C. S.; Bull, J. N.; Curchod, B. F. E.; Verlet, J. R. R. Photoelectron Spectroscopy of the Hexafluorobenzene Cluster Anions: $(C_6F_6)n^-$ (n = 1–5) and $I^-(C_6F_6)$. *The Journal of Physical Chemistry A* **2019**, *123* (8), 1602-1612.

(2) Even, U. "The Even-Lavie valve as a source for high intensity supersonic beam". *EPJ Techniques and Instrumentation* **2015**, *2*, 17.

(3) Wiley, W. C.; McLaren, I. H. Time-of-Flight Mass Spectrometer with Improved Resolution. *Review of Scientific Instruments* **1955**, *26* (12), 1150-1157.

(4) Roberts, G. M.; Nixon, J. L.; Lecointre, J.; Wrede, E.; Verlet, J. R. Toward real-time charged-particle image reconstruction using polar onion-peeling. *Review of Scientific Instruments* **2009**, *80* (5), 053104.

(5) Minnhagen, L. The energy levels of neutral atomic iodine. *Ark. Fys.* **1962**, *21* (26), 415-465.

(6) Dick, B. MELEXIR: maximum entropy Legendre expanded image reconstruction. A fast and efficient method for the analysis of velocity map imaging or photoelectron imaging data. *Physical Chemistry Chemical Physics* **2019**, *21* (35), 19499-19512.

(7) Wigner, E. P. On the Behavior of Cross Sections Near Thresholds. *Physical Review* **1948**, *73* (9), 1002-1009.

(8) Avila Ferrer, F. J.; Santoro, F. Comparison of vertical and adiabatic harmonic approaches for the calculation of the vibrational structure of electronic spectra. *Physical Chemistry Chemical Physics* **2012**, *14* (39), 13549-13563.

(9) Cerezo, J.; Santoro, F. FCclasses3: Vibrationally-resolved spectra simulated at the edge of the harmonic approximation. *Journal of Computational Chemistry* **2023**, *44* (4), 626-643.

(10) Cerezo, J.; Santoro, F. Revisiting Vertical Models To Simulate the Line Shape of Electronic Spectra Adopting Cartesian and Internal Coordinates. *Journal of Chemical Theory and Computation* **2016**, *12* (10), 4970-4985.

(11) Chai, J. D.; Head-Gordon, M. Long-range corrected hybrid density functionals with damped atom-atom dispersion corrections. *Physical Chemistry Chemical Physics* **2008**, *10* (44), 6615-6620.

(12) Peterson, K. A.; Woon, D. E.; Dunning, T. H. Benchmark calculations with correlated molecular wave functions. IV. The classical barrier height of the $H+H_2 \rightarrow H_2+H$ reaction. *The Journal of Chemical Physics* **1994**, *100* (10), 7410-7415.

(13) Frisch, M. J.; Trucks, G. W.; Schlegel, H. B.; Scuseria, G. E.; Robb, M. A.; Cheeseman, J. R.; Scalmani, G.; Barone, V.; Petersson, G. A.; Nakatsuji, H.; et al. Gaussian 16 Rev. C.01. Wallingford, CT, 2016.

(14) Hattig, C.; Tew, D. P.; Kohn, A. Communications: Accurate and efficient approximations to explicitly correlated coupled-cluster singles and doubles, CCSD-F12. *The Journal of Chemical Physics* **2010**, *132* (23), 231102.

(15) Bachorz, R. A.; Bischoff, F. A.; Gloss, A.; Hattig, C.; Hofener, S.; Klopper, W.; Tew, D. P. The MP2-F12 method in the Turbomole program package. *Journal of Computational Chemistry* **2011**, *32* (11), 2492-2513.

(16) Furche, F.; Ahlrichs, R.; Hättig, C.; Klopper, W.; Sierka, M.; Weigend, F. Turbomole. *WIREs Computational Molecular Science* **2013**, *4* (2), 91-100.

(17) Neese, F. The ORCA program system. *WIREs Computational Molecular Science* **2011**, *2* (1), 73-78.

(18) Neese, F. Software Update: The ORCA Program System—Version 6.0. *WIREs Computational Molecular Science* **2025**, *15* (2), e70019.
